# Molecular Pathways Underlying Neurodegenerative and Vascular Pathologies in Alzheimer's Disease

**DOI:** 10.1002/alz70855_107579

**Published:** 2025-12-29

**Authors:** Annie J. Lee, Minghua Liu, David A. A. Bennett, Richard Mayeux, Badri N. Vardarajan

**Affiliations:** ^1^ Department of Neurology, Taub Institute for Research on Alzheimer's Disease and the Aging Brain, Columbia University, New York, NY, USA; ^2^ Columbia University Irving Medical Center, New York, NY, USA; ^3^ Rush Alzheimer's Disease Center, Rush University Medical Center, Chicago, IL, USA; ^4^ Departments of Neurology, Psychiatry, and Epidemiology, Gertrude H. Sergievsky Center, The Taub Institute for Research on Alzheimer's Disease and the Aging Brain, Vagelos College of Physicians and Surgeons, Columbia University, New York, NY, USA; ^5^ Department of Neurology, The Taub Institute for Research on Alzheimer's Disease and The Aging Brain, Columbia University Medical Center and The New York Presbyterian Hospital, New York, NY, USA

## Abstract

**Background:**

Over 70% of Alzheimer's Disease (AD) patients exhibit cerebrovascular pathology, highlighting the complex interaction between neurodegenerative and vascular mechanisms. Understanding the distinct and shared molecular pathways among these pathologies is crucial for advancing our understanding of AD progression.

**Method:**

Participants from the Religious Orders Study/Memory and Aging Project (ROS/MAP) were previously classified into three subgroups: Neurodegenerative, Vascular, and Mixed. We analyzed 1,092 transcriptomic profiles from the prefrontal cortex to identify differentially expressed genes (DEGs) across three comparisons: neurodegenerative vs. vascular, neurodegenerative vs. mixed, and vascular vs mixed. Depending on differential expressions in each of the three categories, we defined genes to be involved purely in neurodegenerative or vascular pathologies. Pathway enrichment analysis was conducted to identify the distinct biological mechanisms underlying these gene groups.

**Result:**

Pathway analysis revealed distinct molecular mechanisms for each group. Genes involved purely in neurodegenerative pathology were enriched in pathways related to heterochromatin formation (e.g., *RESF1, SETDB2, CTCF, EZH2*), epigenetic regulation (e.g., *RESF1, SETDB2, EP300*), and histone modification (e.g., *ATXN7, SETDB2*), essential for gene expression regulation and neuronal function in AD. Genes involved purely in vascular pathology were enriched in mitochondrial processes, including ATP synthesis (e.g., *DNAJC30, NDUFB8, NDUFA6*), cellular respiration (e.g., *DNAJC30, COX3*), and oxidative phosphorylation (e.g., *COX3, NDUFB8*), critical for energy production in vascular pathways. Genes involved purely in mixed pathology were linked to lipid modification, DNA metabolism, proteasome‐mediated degradation, phosphatidylinositol dephosphorylation, and calcium ion response, associated with cellular stress and metabolic regulation. These pathways were largely independent, with minimal overlap.

**Conclusion:**

We identified distinct pathways associated with AD‐related genes and vascular genes, highlighting divergent biological mechanisms driving disease progression. These insights may guide the development of targeted therapeutic strategies for AD with varying vascular involvement.

